# Pregnancy loss and the risk of rheumatoid arthritis in Chinese women: findings from the China Kadoorie biobank

**DOI:** 10.1186/s12889-022-14163-z

**Published:** 2022-09-17

**Authors:** Jia Yi Hee, Sha Huang, Khai Pang Leong, Li Chun, Yuxun Oswald Zhang, Ruofan Gongye, Kun Tang

**Affiliations:** 1 Vanke School of Public Health, Tsinghua University, Zhongguancun North Street, Beijing, 100084 Haidian District China; 2 Department of Rheumatology, Allergy and Immunology, Tan Tock Seng Hospital, Singapore, Singapore; 3 Department of Rheumatology and Immunology, Peking University People’s Hospital, Beijing, China; 4 Department of Maternal and Child Health, Gillings School of Public Health, University of North Carolina, Chapel Hill, NC USA

**Keywords:** Pregnancy loss, Induced abortion, Spontaneous abortion, Stillbirth, Rheumatoid arthritis, China Kadoorie biobank

## Abstract

Considering the female preponderance of rheumatoid arthritis (RA), and disease onset typically after the reproductive years, pregnancy and childbirth may play a role in the aetiology of the disease. Adverse outcomes of pregnancy have been found to precede the diagnosis of autoimmune diseases, including RA, but the evidence is scant and inconsistent. Therefore, we investigate whether pregnancy loss is associated with the risk of RA in Chinese women. Data from the China Kadoorie Biobank, conducted by the University of Oxford and the Chinese Centre for Disease Control and Prevention, of 299,629 Chinese women who had been pregnant were used. Multivariable logistic regression and stratified analyses were employed to analyse the association between types of pregnancy loss with the risk of RA. Pregnancy loss was significantly associated with increased risk of RA (OR 1.12, 95% CI 1.06–1.18), specifically, spontaneous (OR 1.11, 95% CI 1.03–1.20) and induced abortions (OR 1.11, 95% CI 1.06–1.17). There was no significant association between stillbirth and the risk of RA (OR 1.07, 95% CI 0.97–1.18). The risk of developing RA increases with the number of pregnancy losses: one loss confers an OR of 1.09 (95% CI 1.03–1.16), two an OR of 1.13 (95% CI 1.05–1.20), three or more an OR of 1.19 (95% CI 1.10–1.28) and OR of 1.06 (95% CI 1.03–1.08) for each additional. Spontaneous and induced abortions are associated with an increased risk of RA in Chinese women.

**Supplementary Information:**

The online version contains supplementary material available at 10.1186/s12889-022-14163-z.

## Background

Rheumatoid arthritis (RA), a chronic and progressive systemic autoimmune disease, is characterized by symmetric joint inflammation. RA also affects extra-articular organs such as the heart, lungs, and kidneys. It is present in all populations and affects all ages, though its prevalence increases with age [1, 2]. It has been estimated that genetics factors such as human leukocyte antigen alleles accounts for 50% of RA risk factors [3]. Environmental factors such as diet, air-borne exposures, hormones and pregnancy have also been identified to be associated with RA [4].

Akin to many other autoimmune diseases, there is a female preponderance in RA [5]. RA is characterized by an approximate 5:1 female to male ratio [6]. Sex hormones, exogenous (e.g. hormonal contraceptives) and endogenous (e.g. menstruation, pregnancy, and menopause), are believed to be the main cause of this [7]. RA symptoms are reduced during the postovulatory phase of the menstrual cycle, [8] and RA often remits during pregnancy but relapses after delivery [9–12].

It has been hypothesized that adverse pregnancy outcomes are associated with subsequent RA onset, although epidemiological studies have demonstrated conflicting results [13–16]. The first study to investigate reproduction and the onset of RA reported subfertility in women both before and after the onset of RA [13]. Other studies demonstrated that women with RA had higher incidence of spontaneous abortion and stillbirths compared to controls before the onset of disease [15, 17]. These studies suggest the presence of “rheumatic diathesis”, which hypothesizes that the subclinical signs of RA may long antedate the symptoms of the disease [18]. However, a study in newly diagnosed RA patients reported no statistically significant differences in any adverse pregnancy outcomes including spontaneous abortions and stillbirths before the onset of RA [16].

Studies of other autoimmune diseases suggest that adverse pregnancy outcomes may precede the diagnosis of autoimmune disease [19, 20]. Gleicher and el-Roeiy (1999) reported that abnormally high autoantibody levels have the pathophysiological ability to prevent a successful pregnancy, which is speculated to be a measure against the transmission of autoimmunity genes to the next generation [21]. Similarly in RA, the evolutionary situation may prevent successful reproduction in the attempt to reduce the genetic predisposition to RA to the next generation.

As the evidence of the association between adverse pregnancy outcomes and the risk of subsequent RA onset is inconsistent, and that adverse pregnancy outcomes may herald an impending RA diagnosis, the objective of this study is to investigate the association between pregnancy loss and the type of pregnancy loss, with the risk of RA. We hypothesize that the prior occurrence of adverse pregnancy outcomes is associated with increased risk of RA onset in the Chinese population. To our knowledge, no study on adverse pregnancy outcomes and the risk of RA onset in the population of China has been conducted.

## Methods

### Study settings and participants

This cross-sectional study utilizes data from the China Kadoorie Biobank (CKB), a large prospective database initiated by the University of Oxford and the Chinese Centre for Disease Control and Prevention. The aim of the CKB is to recruit, assess, and follow the health of 0.5 million Chinese over the timespan of at least 20 years. The study design and methods of the CKB database have previously been described in detail elsewhere [22, 23].

Briefly, between 2004 to 2008, 302,510 women and 210,205 men from five urban (Qingdao, Harbin, Haikou, Suzhou, and Liuzhou) and five rural (Sichuan, Gansu, Henan, Zhejiang, and Hunan) areas of China, chosen accordingly to local disease patterns, exposure to risk factors of interest, population stability, quality of local disease and death registries, and local commitment and capacity, were recruited [22]. The CKB database has been given ethics approval by the University of Oxford, the Chinese Centre for Disease Control and Prevention (CDC), and the institutional research boards of the local CDCs in the study areas.

Inclusion criteria for the CKB database included eligible participants selected for the study within each region through official residential records, selected participants in possession of a unique national identity card, and selected participants aged between 35 to 74 years. The inclusion criteria for the purposes of our study are female participants of the CKB database who have a history of pregnancy. All participants have provided written informed consent according to the Declaration of Helsinki for participation and to allow for access to their medical records [22].

**Fig. 1:**
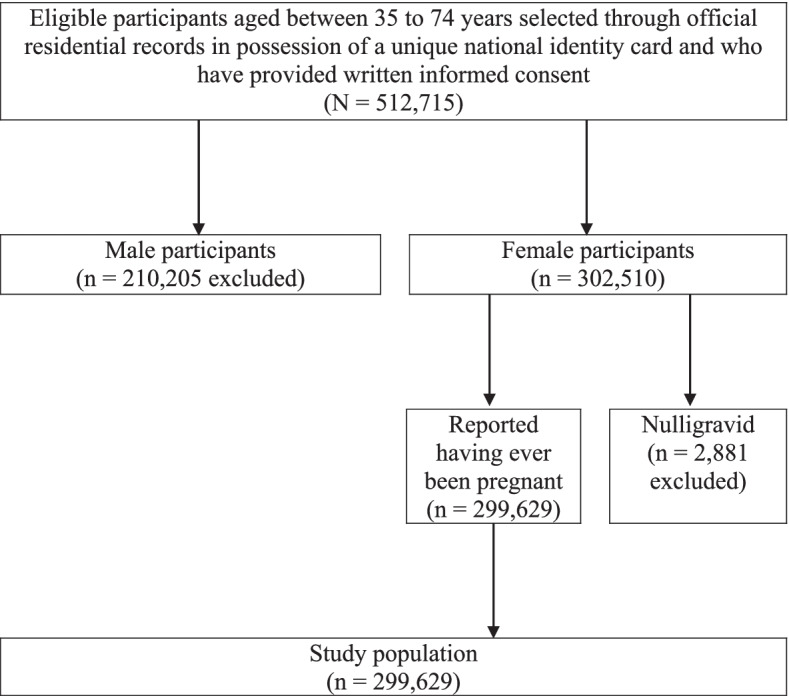
Flow diagram of inclusion or exclusion of study participants

The flow diagram of the inclusion or exclusion of participants is presented in Fig. 1. From an initial cohort of 512,715 participants, 210,205 male participants and 2881 female participants who reported they have never been pregnant were excluded. The remaining 299,629 participants with a history of pregnancy were included in the final analysis.

### Data collection

At the local community assessment centre in each study area, trained medical staff with previous research experience administered an electronic questionnaire that included, but was not limited to, sociodemographic status, dietary and lifestyle habits, medical history, physical activity, and reproductive history of women [22]. Physical measurements such as standing height and weight were also collected by trained technicians according to standard protocol [22]. Repeated sampling of selected items of the questionnaire and physical measurements was carried out at random in approximately 3% of participants from each community to ensure the quality of the data [22].

### Variables of interests

The outcome of interest for this study was the diagnosis of RA. RA was self-reported and include diagnoses made by both physicians and traditional Chinese medicine (TCM) practitioners. The exposures of interest are previous history of pregnancy losses, including spontaneous abortion, induced abortion, and stillbirth. Any experiences of pregnancy loss were self-reported. All types of pregnancy losses; spontaneous abortion, induced abortion, and stillbirth, including total pregnancy loss, were re-categorized into 1, 2 and 3 or more. Other variables of interests include, but are not limited to, region (urban and rural), household income (< 5000 yuan, 5000–19,999 yuan, and ≥ 20,000 yuan), metabolic equivalent of task value (MET) (categorize as below or above the median: < 16.8 and ≥ 16.8), body mass index (BMI) (< 25 and ≥ 25), smoking (smoker and non-smoker), and alcohol consumption (alcohol drinker and non-alcohol drinker).

### Statistical analysis

Categorical variables were described as proportion (percentage), and continuous variables were described as mean ± standard deviation for variables with normal distribution, or as median (interquartile range) for variables with skewed distribution. Categorical variables were compared using the chi-square test. Continuous variables were compared using the one-way analysis of variance (ANOVA) test for variables with normal distribution and the Kruskal-Wallis test for variables with skewed distribution. Logistic regression was performed to obtain the odds ratio (OR) and 95% confidence intervals (95% CI) for the association between total pregnancy loss, spontaneous abortion, induced abortion, and stillbirth with the risk of RA. The models were adjusted for age, province, education, occupation, income, MET, BMI, alcohol consumption, smoking, gum bleed, hypertension diagnosis, diabetes diagnosis, livebirths, and stillbirths, spontaneous abortion, and induced abortion, as appropriate. Collinearity and goodness-of-fit were assessed using variance inflation factors and Hosmer-Lemeshow test. Subgroup analyses were also performed to obtain the ORs and 95% CIs for the risk of RA as associated with pregnancy loss by region, income, MET, BMI, smoking, and alcohol consumption. Missing values were treated and reported as missing in Table 1. Whilst only the results of subgroup analyses between pregnancy loss and the risk of RA were reported, the associations between the types of pregnancy losses; spontaneous abortion, induced abortion, and stillbirth were also assessed (Supplementary Table 2). The level of statistical significance was set at 5% (*p* < 0.05) for all statistical analyses. All statistical modelling excluded nulligravid women and were performed using Stata version 16.0 (College Station, Texas 77,845, USA).

**Table 1:** Characteristic of participants (*N* = 302,510)

	Participants with a history of pregnancy								
	Total	Nulligravid	Total	0	1	2	≥3	Missing	*P*-value
**Number of females participants**	302,510 (100.00)	2,881 (0.95)	299,629 (100.00)	115,283 (38.48)	93,409 (31.18)	57,049 (19.04)	33,838 (11.30)	50 (0.02)	-
**Age, median (IQR)**	50.92 (42.60 – 58.78)	49.98 (40.88 – 59.56)	50.93 (42.62 – 58.77)	51.49 (42.43 – 59.43)	50.68 (42.56 – 58.55)	50.32 (42.66 – 57.91)	50.91 (43.26 – 58.18)	51.54 (40.00 – 61.97)	0.0001
**RA, n (%)**									
No	294,935 (97.50)	2,810 (97.54)	292,125 (97.50)	112,806 (97.85)	91,023 (97.45)	55,449 (97.20)	32,798 (96.93)	49 (98.00)	<0.0001
Yes	7,575 (2.50)	71 (2.46)	7,504 (2.50)	2,477 (2.15)	2,386 (2.55)	1,600 (2.80)	1,040 (3.07)	1 (2.00)	
**Age at RA diagnosis, median (IQR)**	45.00 (37.00 – 53.00)	40.00 (35.00 – 56.00)	45.00 (37.00 – 53.00)	46.00 (38.00 – 53.00)	45.00 (37.00 – 53.00)	45.00 (37.00 – 52.00)	45.00 (37.00 – 52.00)	63.00 (63.00 – 63.00)	0.0115
**Region, n (%)**									
Urban	134,828 (44.57)	1,755 (60.92)	133,073 (44.41)	36,400 (31.57)	46,732 (50.03)	32,222 (56.48)	17,696 (52.30)	23 (46.00)	<0.0001
Rural	167,682 (55.43)	1,126 (39.08)	166,556 (55.59)	78,883 (68.43)	46,677 (49.97)	24,827 (43.52)	16,142 (47.70)	27 (54.00)	
**Education, n (%)**									
Primary or below	171,580 (56.72)	1,248 (43.32)	170,332 (56.85)	76,851 (66.66)	49,867 (53.39)	26,863 (47.09)	16,722 (49.42)	29 (58.00)	<0.0001
Secondary	117,491 (38.84)	1,225 (42.52)	116,266 (38.80)	35,272 (30.60)	38,854 (41.60)	26,792 (46.96)	15,330 (45.30)	18 (36.00)	
Tertiary	13,439 (4.44)	408 (14.16)	13,031 (4.35)	3,160 (2.74)	4,688 (5.02)	3,394 (5.95)	1,786 (5.28)	3 (6.00)	
**Occupation, n (%)**									
Agriculture	122,672 (40.55)	747 (25.93)	121,925 (40.69)	59,585 (51.69)	32,916 (35.24)	17,627 (30.90)	11,772 (34.79)	25 (50.00)	<0.0001
Factory	32,160 (10.63)	336 (11.66)	31,824 (10.62)	9,823 (8.52)	12,477 (13.36)	6,869 (12.04)	2,646 (7.82)	9 (18.00)	
Administrative/Managerial/Sales	19,093 (6.31)	274 (9.51)	18,819 (6.28)	4,839 (4.20)	6,822 (7.30)	4,644 (8.14)	2,510 (7.42)	4 (8.00)	
Professional/ Technical	7,960 (2.63)	224 (7.78)	7,736 (2.58)	1,890 (1.64)	2,684 (2.87)	2,027 (3.55)	1,135 (3.35)	0 (0.00)	
Unemployed/Retired	61,773 (20.42)	773 (26.83)	61,000 (20.36)	15,860 (13.76)	21,093 (22.58)	15,501 (27.17)	8,537 (25.23)	9 (18.00)	
Housewife	47,578 (15.73)	432 (14.99)	47,146 (15.73)	20,013 (17.36)	13,589 (14.55)	7,879 (13.81)	5,664 (16.74)	1 (2.00)	
Self-employed/Others	11,274 (3.73)	95 (3.30)	11,179 (3.73)	3,273 (2.84)	3,282 (4.10)	2,502 (4.39)	1,574 (4.65)	2 (4.00)	
**Marital status, n (%)**									
Married	269,166 (88.98)	1,902 (66.02)	267,264 (89.20)	102,986 (89.33)	83,549 (89.44)	50,950 (89.31)	29,732 (87.87)	47 (94.00)	<0.0001
Widowed	28,069 (9.28)	293 (10.17)	27,776 (9.27)	11,301 (9.80)	8,360 (8.95)	4,919 (8.62)	3,194 (9.44)	2 (4.00)	
Separated/ Divorced	4,573 (1.51)	105 (3.64)	4,468 (1.49)	969 (0.84)	1,458 (1.56)	1,148 (2.01)	892 (2.64)	1 (2.00)	
Single	702 (0.23)	581 (20.17)	121 (0.04)	27 (0.02)	42 (0.04)	32 (0.06)	20 (0.06)	0 (0.00)	
**Household income (yuan), n (%)**									
<5000	30,720 (10.16)	348 (12.08)	30,372 (10.14)	15,998 (13.88)	7,487 (8.02)	4,035 (7.07)	2,847 (8.41)	5 (10.00)	<0.0001
5000 – 19,999	148,702 (49.16)	1,507 (52.31)	147,195 (49.13)	60,208 (52.23)	42,622 (45.63)	26,853 (47.07)	17,487 (51.68)	25 (50.00)	
≥20,000	123,088 (40.69)	1,026 (35.61)	122,062 (40.74)	39,077 (33.90)	43,300 (46.36)	26,161 (45.86)	13,504 (39.91)	20 (40.00)	
**Waist-hip ratio, mean (SD)**	0.87 (0.07)	0.85 (0.08)	0.87 (0.07)	0.87 (0.07)	0.86 (0.07)	0.86 (0.07)	0.87 (0.07)	0.87 (0.07)	<0.0001
**MET hours, n (%)**									
<16.8	149,833 (49.53)	1,683 (58.42)	148,150 (49.44)	53,272 (46.21)	46,318 (49.59)	29,983 (52.56)	18,553 (54.83)	24 (48.00)	<0.0001
≥16.8	152,677 (50.47)	1,198 (41.58)	151,479 (50.56)	62,011 (53.79)	47,091 (50.41)	27,066 (47.44)	15,285 (45.17)	26 (52.00)	
**BMI, n (%)**									
<25	198,798 (65.72)	1,989 (69.04)	196,809 (65.68)	77,486 (67.21)	61,090 (65.40)	36,615 (64.18)	21,580 (63.77)	38 (76.00)	<0.0001
≥25	103,711 (34.28)	892 (30.96)	102,819 (34.32)	37,797 (32.79)	32,319 (34.60)	20,433 (35.82)	12,258 (36.23)	12 (24.00)	
Missing	1 (0.00)	0 (0.00)	1 (0.00)	0 (0.00)	0 (0.00)	1 (0.00)	0 (0.00)	0 (0.00)	
**Smoking, n (%)**									
Smoker	15,330 (5.07)	189 (6.56)	15,141 (5.05)	5,213 (4.52)	4,395 (4.71)	3,008 (5.27)	2,511 (7.42)	14 (28.00)	
Non-smoker	287,180 (94.93)	2,692 (93.44)	284,488 (94.95)	110,070 (95.48)	89,014 (95.29)	54,041 (94.73)	31,327 (92.58)	36 (72.00)	<0.0001
**Alcohol, n (%)**									
Alcohol drinker	110,187 (36.42)	1,208 (41.93)	108,979 (36.37)	36,279 (31.47)	34,345 (36.77)	23,504 (41.20)	14,828 (43.82)	23 (46.00)	
Non-alcohol drinker	192,323 (63.58)	1,673 (58.07)	190,650 (63.63)	79,004 (68.53)	59,064 (63.23)	33,545 (58.80)	19,010 (56.18)	27 (54.00)	<0.0001
**Gum bleed, n (%)**									
No	188,458 (62.30)	1,825 (63.35)	186,633 (62.29)	70,790 (61.41)	58,609 (62.74)	35,927 (62.98)	21,270 (62.86)	37 (74.00)	<0.0001
Yes	114,052 (37.70)	1,056 (36.65)	112,996 (37.71)	44,493 (38.59)	34,800 (37.26)	21,122 (37.02)	12,568 (37.14)	13 (26.00)	
**Parity, n (%)**									
0	4,142 (1.37)	-	1,365 (0.46)	104 (0.09)	635 (0.68)	324 (0.57)	302 (0.89)	0 (0.00)	<0.0001
1	105,732 (34.95)	-	105,637 (35.26)	27,422 (23.79)	37,339 (39.97)	25,695 (45.04)	15,163 (44.81)	18 (36.00)	
2	99,339 (32.84)	-	99,330 (33.15)	42,852 (37.17)	29,934 (32.05)	17,036 (29.86)	9,494 (28.06)	14 (28.00)	
≥3	93,261	-	93,261 (31.13)	44,891 (38.94)	25,487 (27.29)	13,988 (24.52)	8,877 (26.23)	18 (36.00)	
Missing	36 (0.01)	-	36 (0.01)	14 (0.01)	14 (0.01)	6 (0.01)	2 (0.01)	0 (0.00)	
**Pregnancy, n (%)**									
0	2,881 (0.95)	-	-	-	-	-	-	-	
1	27,386 (9.05)	-	27,386 (9.14)	26,765 (23.22)	620 (0.66)	0 (0.00)	0 (0.00)	1 (2.00)	<0.0001
2	78,036 (25.80)	-	78,036 (26.04)	40,834 (35.43)	36,885 (39.49)	308 (0.54)	0 (0.00)	0 (0.00)	
≥3	194,159 (64.18)	-	194,159 (64.80)	47,675 (41.35)	55,903 (59.85)	56,741 (99.46)	33,838 (100.00)	2 (4.00)	
Missing	48 (0.02)	-	48 (0.02)	0 (0.00)	1 (0.00)	0 (0.00)	0 (0.00)	47 (94.00)	
**Livebirths, n (%)**									
0	1,214 (0.40)	-	1,214 (0.41)	0 (0.00)	620 (0.66)	307 (0.54)	287 (0.85)	0 (0.00)	<0.0001
1	104,047 (34.39)	-	104,047 (34.73)	26,765 (23.22)	36,885 (39.49)	25,437 (44.59)	14,958 (44.20)	2 (4.00)	
2	96,042 (31.75)	-	96,042 (32.05)	40,843 (35.43)	29,036 (31.08)	16,766 (29.39)	9,397 (27.77)	0 (0.00)	
≥3	98,279 (32.49)	-	98,279 (32.80)	47,675 (41.35)	26,868 (28.76)	14,539 (25.49)	9,196 (27.18)	1 (2.00)	
Nulligravid	2,881 (0.95)	-	-	-	-	-	-	-	
Missing	47 (0.02)	-	47 (0.02)	0 (0.00)	0 (0.00)	0 (0.00)	0 (0.00)	47 (94.00)	
**Spontaneous abortion, n (%)**									
0	272,424 (90.05)	-	272,424 (90.92)	115,283 (100.00)	81,454 (87.20)	48,584 (85.16)	27,103 (80.10)	0 (0.00)	<0.0001
1	21,412 (7.08)	-	21,412 (7.15)	0 (0.00)	11,955 (12.80)	5,780 (10.13)	3,676 (10.86)	1 (2.00)	
2	4,240 (1.40)	-	4,240 (1.42)	0 (0.00)	0 (0.00)	2,685 (4.71)	1,555 (4.60)	0 (0.00)	
≥3	1,504 (0.50)	-	1,504 (0.50)	0 (0.00)	0 (0.00)	0 (0.00)	1,504 (4.44)	0 (0.00)	
Nulligravid	2,881 (0.95)	-	-	-	-	-	-	-	
Missing	49 (0.02)	-	49 (0.02)	0 (0.00)	0 (0.00)	0 (0.00)	0 (0.00)	49 (98.00)	
**Induced abortion, n (%)**									
0	142,348 (47.06)	-	142,348 (47.51)	115,283 (100.00)	19,135 (20.49)	5,370 (9.41)	2,560 (7.57)	0 (0.00)	<0.0001
1	83,153 (27.49)	-	83,153 (27.75)	0 (0.00)	74,274 (79.51)	7,211 (12.64)	1,668 (4.93)	0 (0.00)	
2	48,160 (15.92)	-	48,160 (16.07)	0 (0.00)	0 (0.00)	44,468 (77.95)	3,692 (10.91)	0 (0.00)	
≥3	25,919 (8.57)	-	25,919 (8.65)	0 (0.00)	0 (0.00)	0 (0.00)	25,918 (76.59)	1 (2.00)	
Nulligravid	2,881 (0.95)	-	-	-	-	-	-	-	
Missing	49 (0.02)	-	49 (0.02)	0 (0.00)	0 (0.00)	0 (0.00)	0 (0.00)	49 (98.00)	
**Stillbirth, n (%)**									
0	282,538 (93.40)	-	282,538 (94.30)	115,283 (100.00)	86,229 (92.31)	51,914 (91.00)	29,112 (86.03)	0 (0.00)	<0.0001
1	13,174 (4.35)	-	13,174 (4.40)	0 (0.00)	7,180 (7.69)	3,469 (6.08)	2,525 (7.46)	0 (0.00)	
2	2,762 (0.91)	-	2,762 (0.92)	0 (0.00)	0 (0.00)	1,666 (2.92)	1,096 (3.24)	0 (0.00)	
≥3	1,105 (0.37)	-	1,105 (0.37)	0 (0.00)	0 (0.00)	0 (0.00)	1,105 (3.27)	0 (0.00)	
Nulligravid	2,881 (0.95)	-		-	-	-	-	-	
Missing	50 (0.02)	-	50 (0.02)	0 (0.00)	0 (0.00)	0 (0.00)	0 (0.00)	50 (100.00)	

*BMI* Body mass index, *MET* Metabolic equivalent of task value, *RA* Rheumatoid arthritis

## Results

### Participants’ characteristics

The characteristics of the participants are presented in Table 1. Among the 302,510 women, 99.05% (*n* = 299,629) reported having ever been pregnant, of which 9.07% (*n* = 27,156) had a history of spontaneous abortion, 52.47% (*n* = 157,232) had a history of induced abortion, 5.69% (*n* = 17,041) had a history of stillbirth. 2.50% (*n* = 7504) have been diagnosed with RA. The median age of these women at survey and at RA diagnosis were 50.93 (IQR: 42.62–58.77) and 45.00 (IQR: 37.00–53.00), respectively. The age of the majority of women at RA diagnosis was past the age of reproduction, therefore the majority of women developed RA after the occurrence of pregnancy loss. Of the women who reported having ever been pregnant, 61.52% had a history of pregnancy loss, 55.59% resided in rural areas, 56.85% had education of primary education or below, 40.69% were employed in agriculture, 89.20% were married, 49.13% had household incomes between 5000 to 19,999 Chinese yuan, 50.56% had MET of 16.8 hours or more, 65.68% had BMI of less than 25, 94.95% did not smoke and 63.63% did not drink alcohol.

Compared to women without a history of pregnancy loss, those with pregnancy loss were significantly more likely to be diagnosed with RA (2.73% vs. 2.15%), resided in urban regions (52.44% vs. 31.57%), have completed secondary education or above (49.29% vs. 33.34%), have a household income > 20,000 Chinese yuan (45.02% vs. 33.90%), have MET hours 16.8 or less (51.47% vs. 46.21%), have a BMI of 25 or more (35.28% vs. 32.79%), smoked tobacco (5.38% vs. 4.52%) and consumed alcohol (39.43% vs. 31.47%) (Supplementary Table 1).

### Pregnancy loss and the risk of rheumatoid arthritis

**Fig. 2:**
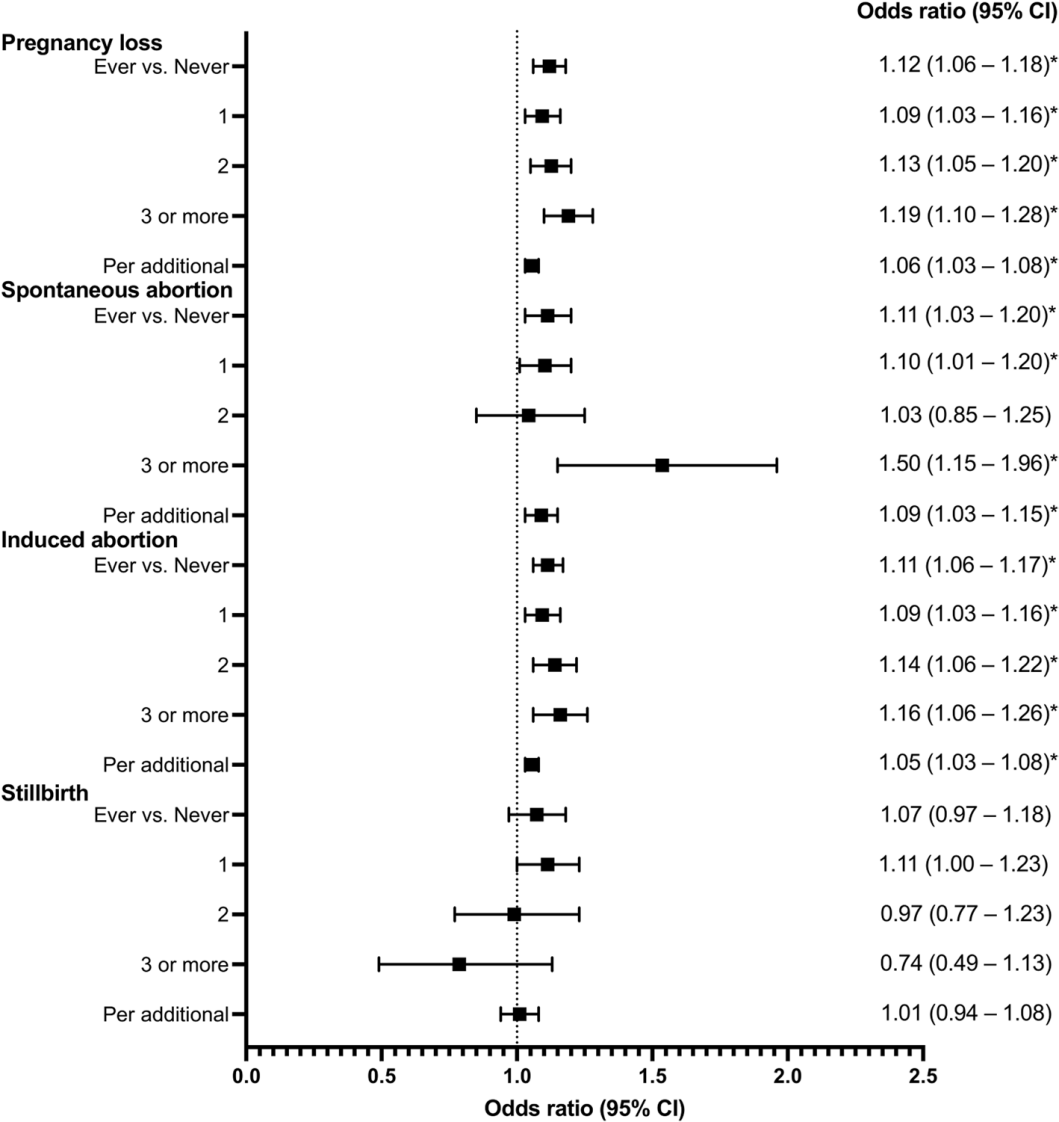
Effect estimates of the association between pregnancy loss and rheumatoid arthritis risk. Adjustments are as in Table 2 model 2

The association between pregnancy loss, including spontaneous abortion, induced abortion, and stillbirth, with the risk of RA is presented in Table 2 and Fig. 2.

**Table 2:** Effect estimates of the association between pregnancy loss and rheumatoid arthritis risk

	No. of events	Model 1	Model 2
**Pregnancy loss**			
Ever vs. never		1.28 (1.22–1.34)*	1.12 (1.06–1.18)*
None	2477	1.00 (reference)	1.00 (reference)
1	2386	1.19 (1.13–1.26)*	1.09 (1.03–1.16)*
2	1600	1.31 (1.23–1.40)*	1.13 (1.05–1.20)*
≥3	1040	1.44 (1.34–1.55)*	1.19 (1.10–1.28)*
Per additional		1.13 (1.11–1.16)*	1.06 (1.03–1.08)*
**Spontaneous abortion**			
Ever vs. never		1.14 (1.05–1.23)*	1.11 (1.03–1.20)*
None	6742	1.00 (reference)	1.00 (reference)
1	593	1.12 (1.03–1.22)*	1.10 (1.01–1.20)*
2	110	1.05 (0.87–1.27)	1.03 (0.85–1.25)
≥3	58	1.58 (1.21–2.06)*	1.50 (1.15–1.96)*
Per additional		1.10 (1.05–1.16)*	1.09 (1.03–1.15)*
**Induced abortion**			
Ever vs. never		1.24 (1.19–1.30)*	1.11 (1.06–1.17)*
None	3174	1.00 (reference)	1.00 (reference)
1	2180	1.18 (1.12–1.25)*	1.09 (1.03–1.16)*
2	1374	1.29 (1.21–1.37)*	1.14 (1.06–1.22)*
≥3	775	1.35 (1.25–1.46)*	1.16 (1.06–1.26)*
Per additional		1.12 (1.09–1.14)*	1.05 (1.03–1.08)*
**Stillbirth**			
Ever vs. never		1.16 (1.06–1.28)*	1.07 (0.97–1.18)
None	7013	1.00 (reference)	1.00 (reference)
1	392	1.20 (1.09–1.34)*	1.11 (1.00–1.23)
2	75	1.10 (0.87–1.38)	0.97 (0.77–1.23)
≥3	23	0.84 (0.55–1.26)	0.74 (0.49–1.13)
Per additional		1.07 (1.01–1.15)*	1.01 (0.94–1.08)

*Statistically significant at the 5% level

Models for pregnancy loss, spontaneous abortion, induced abortion, and stillbirth excludes nulligravid women

**Model 1**: unadjusted

**Model 2:** adjusted for age, province, region, education, occupation, income, metabolic equivalent of task value, body mass index, alcohol use, smoking, gum bleed, hypertension diagnosis, diabetes diagnosis, livebirths, and stillbirths, spontaneous abortion, induced abortion, as appropriate

Compared to women without a history of pregnancy loss, those with pregnancy loss were significantly more likely to be diagnosed with RA, OR 1.12 (95% CI 1.06–1.18). Similarly, women with a history of spontaneous abortion, induced abortion, and stillbirth were more likely to be diagnosed with RA, OR 1.11 (95% CI 1.03–1.20), OR 1.11 (95% CI 1.06–1.17) and OR 1.07 (95% CI 0.97–1.18) respectively, although the association was not significant for women with a history of stillbirth.

An increase in the number of pregnancy losses was significantly associated with increased odds of RA: OR 1.09 (95% CI 1.03–1.16), OR 1.13 (95% CI 1.05–1.20), and OR 1.19, (95% CI 1.10–1.28) for one, two, and three or more pregnancy losses respectively. Similarly, women with increased number of pregnancy losses due to induced abortions were significantly more likely to be diagnosed with RA; OR 1.09 (95% CI 1.03–1.16), OR 1.14 (95% CI 1.06–1.22), and OR 1.16 (95% CI 1.06–1.26) for one, two, and three or more induced abortions respectively. However, this was not apparent in pregnancy losses due to spontaneous abortions (OR 1.10, 95% CI 1.01–1.20, OR 1.03, 95% CI 0.85–1.25, and OR 1.50, 95% CI 1.15–1.96 for one, two, and three or more spontaneous abortions respectively) or stillbirths (OR 1.11, 95% CI 1.00–1.23, OR 0.97, 95% CI 0.77–1.23 and OR 0.74, 95% CI 0.49–1.13 for one, two, and three or more stillbirths respectively).

Each additional pregnancy loss was also associated with increased odds of RA (OR 1.06, 95% CI 1.03–1.08). Similarly, each additional spontaneous abortion, induced abortion, and stillbirth was associated with increased odds of RA, OR 1.09 (95% CI 1.03–1.15), OR 1.05 (95% CI 1.03–1.08), and OR 1.01 (95% CI 0.94–1.08) respectively, although the association was not significant for each additional stillbirth.

### Pregnancy loss and the risk of rheumatoid arthritis, stratified by participants’ characteristics

**Fig. 3:**
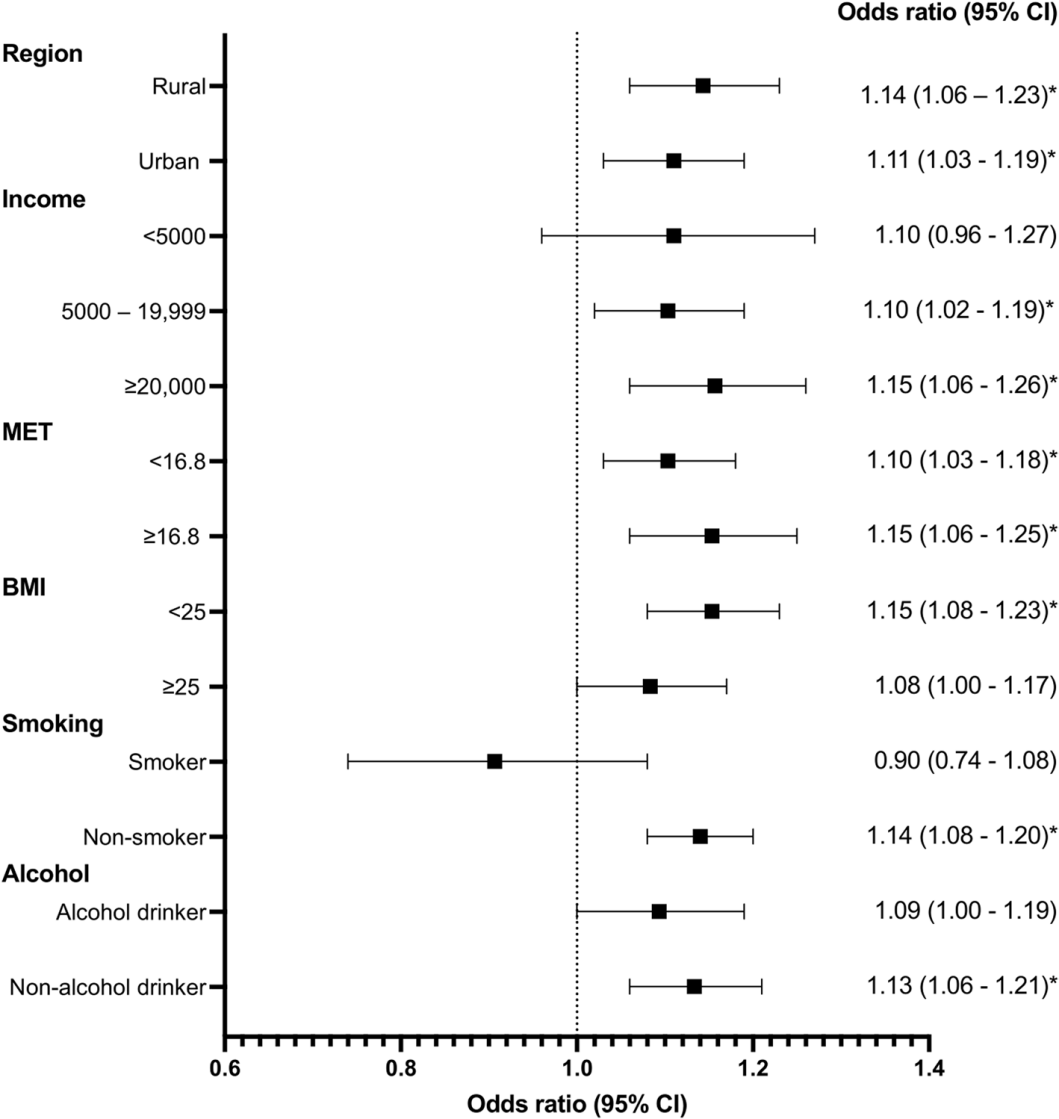
Stratified effect estimates of the association between pregnancy loss (ever vs. never) and rheumatoid arthritis risk. Adjustments are as in Table 3

The associations between pregnancy loss with the risk of RA stratified by region, income, MET, BMI, smoking, and alcohol consumption is presented in Table 3 and Fig. 3.

**Table 3:** Stratified effect estimates of the association between pregnancy loss and rheumatoid arthritis risk

	Pregnancy loss				
	Ever	1	2	≥3	Per additional
**Region**					
Rural	1.14 (1.06–1.23)*	1.12 (1.02–1.22)*	1.13 (1.01–1.26)*	1.23 (1.09–1.40)*	1.07 (1.03–1.11)*
Urban	1.11 (1.03–1.19)*	1.08 (0.99–1.16)	1.12 (1.03–1.22)*	1.17 (1.06–1.29)*	1.05 (1.02–1.09)*
**Income**					
< 5000	1.10 (0.96–1.27)	1.07 (0.91–1.26)	1.01 (0.82–1.25)	1.29 (1.04–1.61)*	1.06 (1.00–1.14)
5000–19,999	1.10 (1.02–1.19)*	1.06 (0.97–1.15)	1.15 (1.04–1.26)*	1.15 (1.03–1.28)*	1.05 (1.02–1.09)*
≥20,000	1.15 (1.06–1.26)*	1.14 (1.03–1.25)*	1.13 (1.02–1.26)*	1.21 (1.07–1.37)*	1.06 (1.02–1.10)*
**MET**					
< 16.8	1.10 (1.03–1.18)*	1.06 (0.98–1.14)	1.12 (1.03–1.22)*	1.17 (1.07–1.29)*	1.06 (1.02–1.09)*
≥16.8	1.15 (1.06–1.25)*	1.14 (1.04–1.25)*	1.13 (1.01–1.26)*	1.20 (1.06–1.37)*	1.06 (1.02–1.10)*
**BMI**					
< 25	1.15 (1.08–1.23)*	1.13 (1.04–1.22)*	1.14 (1.05–1.25)*	1.23 (1.11–1.36)*	1.07 (1.04–1.10)*
≥25	1.08 (1.00–1.17)	1.04 (0.95–1.15)	1.11 (1.00–1.23)	1.14 (1.01–1.28)*	1.05 (1.01–1.09)*
**Smoking**					
Smoker	0.90 (0.74–1.08)	0.82 (0.66–1.03)	0.90 (0.70–1.16)	1.05 (0.81–1.36)	1.01 (0.93–1.10)
Non-smoker	1.14 (1.08–1.20)*	1.11 (1.05–1.18)*	1.14 (1.07–1.23)*	1.19 (1.10–1.29)*	1.06 (1.03–1.09)*
**Alcohol**					
Alcohol drinker	1.09 (1.00–1.19)	1.06 (0.96–1.18)	1.09 (0.98–1.22)	1.14 (1.01–1.29)*	1.04 (1.00–1.08)*
Non-alcohol drinker	1.13 (1.06–1.21)*	1.10 (1.02–1.19)*	1.14 (1.04–1.24)*	1.21 (1.10–1.34)*	1.06 (1.03–1.10)*

*Statistically significant at the 5% level

*BMI* Body mass index, *MET* Metabolic equivalent of task value

Models excludes nulligravid women. Women without pregnancy loss were used as reference

**Region:** Adjusted for age, province, education, occupation, income, physical activity, body mass index, alcohol use, smoking, gum bleed, hypertension diagnosis, diabetes diagnosis, and livebirths

**Income:** Adjusted for age, province, region, education, occupation, physical activity, body mass index, alcohol use, smoking, gum bleed, hypertension diagnosis, diabetes diagnosis, and livebirths

**Metabolic equivalent of task value:** Adjusted for age, province, region, education, occupation, income, body mass index, alcohol, smoking, gum bleed, hypertension diagnosis, diabetes diagnosis, and livebirths

**Body mass index:** Adjusted for age, province, region, education, occupation, income, physical activity, alcohol use, smoking, gum bleed, hypertension diagnosis, diabetes diagnosis, and livebirths

**Smoking:** Adjusted for age, province, region, education, occupation, income, physical activity, body mass index, alcohol use, gum bleed, hypertension diagnosis, diabetes diagnosis, and livebirths

**Alcohol:** Adjusted for age, province, region, education, occupation, income, physical activity, body mass index, smoking, gum bleed, hypertension diagnosis, diabetes diagnosis, and livebirths

There was little heterogeneity in the associations between pregnancy loss (ever vs. never) and the risk of RA by subgroup analyses. Pregnancy loss was positively associated with the risk of RA in all subgroup analyses, except for smokers, for which there was an inverse association. However, an income of < 5000 Chinese yuan (< approximately 780 United States Dollars) (OR 1.10, 95% CI 0.96–1.27), a BMI of ≥25 (OR 1.08, 95% CI 1.00–1.17), smoking (OR 0.90, 95% CI 0.74–1.08) and alcohol consumption (OR 1.09, 95% CI 1.00–1.19) in women with a history of pregnancy loss were not significantly associated with increased risk of RA.

The dose-response relationship between one, two, and three or more pregnancy losses with increased risk of RA was apparent in women regardless of their region of residence, BMI, smoking and alcohol statuses, as well as in women with MET hours of less than 16.8. However, the dose-response relationship was not significant in women with a BMI of ≥25 (OR 1.04, 95% CI 0.95–1.15, OR 1.11, 95% CI 1.00–1.23, and OR 1.14, 95% CI 1.01–1.28 respectively), smokers (OR 0.82, 95% CI 0.66–1.03, OR 0.90, 95% CI 0.70–1.16, and OR 1.05, 95% CI 0.81–1.36 respectively), and alcohol drinkers (OR 1.06, 95% CI 0.96–1.18, OR 1.09, 95% CI 0.98–1.22, and OR 1.14, 95% CI 1.01–1.29, respectively).

There was also little heterogeneity in the associations between each additional pregnancy loss and the risk of RA by subgroup analyses. Each additional pregnancy loss was positively associated with the risk of RA in all subgroup analyses. However, this association was not significant in women with an income of < 5000 Chinese yuan (OR 1.06, 95% CI 1.00–1.14) and smokers (OR 1.01, 95% CI 0.93–1.10).

## Discussion

In this cohort of Chinese women, pregnancy loss, spontaneous or induced, was associated with increased risk of RA. Stillbirth, however, was not significantly associated with a risk of RA. Increased number of pregnancy losses due to induced abortion and each additional spontaneous or induced abortion were also found to be associated with increased risk of RA.

Similar to our findings, another group found a higher incidence of abortion in women with RA, before disease onset [17]. However, other studies have reported no significant association between pregnancy losses, including spontaneous and induced abortion, and the subsequent risk of RA [24, 25].

There are two hypotheses to explain the association of pregnancy loss and RA: (1) foetal cells enter the mother’s circulatory system before pregnancy loss, and (2) due to infections associated with pregnancy loss.

The first theory proposes that genetically disparate cells associated with risk of RA of foetal origin are transferred from the foetus to their mother. This is known as foetal microchimerism. The risk of subsequent RA onset was found to be increased in women who had a child with an allele encoded with the share epitope (human leukocyte antigen-DRB1 [HLA-DRB1] alleles encoding QKRAA, QRRAA or RRRAA amino acid sequence at positions 70–74 of the HLA-DRβ1 chain [26]). The presence of HLA-DRB1 allele encoded with DERAA sequence, while usually RA-protective, was found to have increase the odds of RA by approximately 17 times in women with DERAA-positive children born prior to RA onset, suggesting that the RA protective sequence when acquired through microchimerism, is harmful [27].

During pregnancy, there is bidirectional exchange of cells between the mother and foetus which may persist in the maternal circulation for decades after the pregnancy [28]. These cells or deoxyribonucleic acid (DNA) that are genetically disparate can affect the long-term health of mothers beneficially or adversely [28]. Foetal microchimerism is considered by Nelson and Lambert (2017) to be akin to “reverse inheritance” [28, 29]. Foetal microchimeric cells contribute to the development of RA by either being targets for immune response or by working as effector cells [30]. Conversely, foetal microchimeric cells can also be beneficial to the prevention of RA by contributing to tissue repair and regeneration [30]. This is because foetal microchimeric cells can differentiate into tissue specific phenotypes [30].

Spontaneous abortions and induced abortions have also been known to produce foetal origin microchimerism, although the composition of microchimerism is likely to be different from those of livebirths due to changes in the cell types over the course of gestation [31–33]. In women without sons, male DNA (commonly used to test for bidirectional exchange of cells as female DNA would be naturally present in the mother) was found in the peripheral blood in almost a quarter of women who had spontaneous abortions, and more than half who had induced abortion [31]. Only pregnancy loss was reportedly significantly associated with the presence of foetal microchimerism [34]. Higher levels of foetal microchimerism were also found in women with a trisomy 21 foetus as compared to women with a normal foetus [35], suggesting that offspring with genetic anomalies are more likely to contribute to foetal microchimerism. Foetuses with genetic anomalies are also more likely to be spontaneously or medically aborted. Pregnancies with poor outcomes have been associated with increased risk of poor outcomes in subsequent pregnancies [36]. This may explain the association between spontaneous and induced abortions with risk of RA and the dose-response relationship of the number of pregnancy losses.

The second theory posits that infections associated with pregnancy loss contributes to the development of RA. Foetal tissue, which are typically naturally delivered from the uterus after childbirth, may be retained in the uterus in cases of spontaneous abortions or induced abortions [37]. During the one-child policy in China, women were required to have an intrauterine device (IUD) fitted after their first birth [38]. It has been reported that approximately 70% of the estimated 11 million induced abortions performed per year were mainly due to IUD failure [38] and that pregnancy while using an IUD result in 5-fold higher risk of spontaneous abortion [39]. Studies have suggested that retained products of conception occurs in more than 40% of abortions during the first and second trimester [40, 41] and that infections following abortions mainly result from the infection of products of conception retained in the uterine cavity [42]. Retained products of conception, or surgery to removed retained products of conception (typically hysteroscopy or dilation and curettage), have also been associated with risk of infections [42].

Several pathogenic mechanisms of infection on RA have been proposed. Endogenous citrullinated proteins from certain bacteria can citrullinate human proteins such as common RA antigens fibrinogen and a-enolase [43]. However, this has only been established in *P. gingivalis*, a bacteria involved in the pathogenesis of periodontitis [43]. Bacteria may also be able to mimic self-proteins, triggering autoantibody production through epitope spreading [44]. It has also been reported that microbial infection can directly damage joints by contributing to cartilage loss and bone destruction [45, 46].

We found several findings that were unexpected. First, we found a high prevalence of RA in this cohort of women and the sample population. This was unexpected as China reportedly has one of the lowest RA prevalence in the world, with an estimated prevalence of 0.42% [47]. For comparison, the total prevalence of RA in the entire cohort including men and nulligravid women was 2.07% (*n* = 10,623). This may be so as diagnoses of RA were self-reported and include those made by both physicians and TCM practitioners. Second, there was a lack of a significant association between stillbirth and the risk of RA contrary to the outcome of another study that RA patients were 12.5 times more likely to have experienced a stillbirth [15]. A possible reason for our finding is that RA autoantibodies present in the mother or genetical anomalies in the fetus prevents the body or the fetus from advancing past the 20th week in the pregnancy, causing the pregnancy to be terminated earlier on as spontaneous or induced abortion. Last, there was the lack of association between pregnancy loss and RA in women who are obese, smokers, and alcohol drinkers. As these women are known to have subfecundity, the association between pregnancy loss and the risk of RA may be masked [48–50]. It is also possible that whilst we adjusted for confounders, there could have been residual confounding or confounding from variables not collected.

Overall, our study has several strengths. First, our study had a much larger sample size of women compared to all the previous studies conducted. Second, the comprehensive data collected allowed for the analysis of the various types of pregnancy losses on the risk of RA. Third, as the data collection was conducted across various areas in China, our study may have better representativity which allows for better generalizability of our findings. Last, all participants were extensively interviewed by specifically trained interviewers to collect their medical history, as well as their history of pregnancy and pregnancy losses, to ensure data quality.

Our study also has several limitations. First, the areas selected for data collection were based on various factors such as the quality of local registries and long-term local commitment, rather than random selection. However, this is to ensure that areas with different disease profiles and exposures will be covered, and that there is sufficient participation. Second, given the cross-sectional nature of this study, we were unable to determine the causality between pregnancy loss and RA. Third, given that the data collection was conducted between 2004 to 2008, the findings from this study may not be as relevant to the population at present. However, the time period of 2004 to 2008 is a unique time period of importance that should be studied, as it overlaps with the period of the one-child policy implementation. Given the high rates of multiple induced abortions (51.98%), it allowed for the study of the dose-response relationship between pregnancy loss and the risk of RA. Last, as the questionnaire mainly utilizes self-reporting, we rely upon participants’ recollection which may result in recall bias.

## Conclusion

Our findings show that spontaneous and induced abortions are significantly positively associated with the risk of RA in Chinese women. Pregnancy loss and RA are represented by a complex spectrum of other biological and non-biological factors and further research needs to be conducted to improve our understanding on the relation between pregnancy loss and RA.

## Supplementary Information

**Additional file 1.**

## Data Availability

The data underlying this article were provided by the China Kadoorie Biobank by permission. Data will be shared on request to the corresponding author with permission of *the China* Kadoorie Biobank.

## Abbreviations

ANOVA: Analysis of variance
BMI: Body mass index
CDC: Centre for Disease Control and Prevention
CI: Confidence interval
CKB: China Kadoorie Biobank
DNA: Deoxyribonucleic acid
HLA-DRB1: Human leukocyte antigen-DRB1
IUD: Intrauterine device
MET: Metabolic equivalent of task value
OR: Odds ratio
RA: Rheumatoid arthritis
TCM: Traditional Chinese medicine
