# Supplementary material for: Pregnancy loss and the risk of rheumatoid arthritis in Chinese women: findings from the China Kadoorie biobank
Source: BMC Public Health. 2022 Sep 17;22:1768. doi: 10.1186/s12889-022-14163-z (PMC9482729; doi:10.1186/s12889-022-14163-z)
Supplement: Supplementary file 1 — Additional file 1. [file 12889_2022_14163_MOESM1_ESM.docx]

**Supplementary material**

Table 1. Characteristic of participants according to pregnancy loss (N = 299,629)

|  |  | **Number of pregnancy losses (n = 299,629)** | | |  |
| --- | --- | --- | --- | --- | --- |
|  | Total (n = 299,629) | No pregnancy loss | Had pregnancy loss | Missing | P-value |
| **Number of females** | 299,629 (100.00) | 115,283 (38.48) | 184,296 (61.51) | 50 (0.02) |  |
| **Age, median (IQR)** | 50.93 (42.62 – 58.77) | 51.49 (42.43 – 59.43) | 50.61 (42.72 – 58.30) | 51.54 (40.00 – 61.97) | 0.0001 |
| **RA, n (%)** |  |  |  |  |  |
| No | 292,125 (97.50) | 112,806 (97.85) | 179,270 (97.27) | 49 (98.00) | <0.0001 |
| Yes | 7,504 (2.50) | 2,477 (2.15) | 5,026 (2.73) | 1 (2.00) |  |
| **Age at RA diagnosis, median (IQR)** | 45.00 (37.00 – 53.00) | 46.00 (38.00 – 53.00) | 45.00 (37.00 – 52.00) | 63.00 (63.00 – 63.00) | 0.0166 |
| **Region, n (%)** |  |  |  |  |  |
| Urban | 133,073 (44.41) | 36,400 (31.57) | 96,650 (52.44) | 23 (46.00) | <0.0001 |
| Rural | 166,556 (55.59) | 78,883 (68.43) | 87,646 (47.56) | 27 (54.00) |  |
| **Education, n (%)** |  |  |  |  |  |
| Primary or below | 170,332 (56.85) | 76,851 (66.66) | 93,452 (50.71) | 29 (58.00) | <0.0001 |
| Secondary | 116,266 (38.80) | 35,272 (30.60) | 80,976 (43.94) | 18 (36.00) |  |
| Tertiary | 13,031 (4.35) | 3,160 (2.74) | 9,868 (5.35) | 3 (6.00) |  |
| **Occupation, n (%)** |  |  |  |  |  |
| Agriculture | 121,925 (40.69) | 59,585 (51.69) | 62,315 (33.81) | 25 (50.00) | <0.0001 |
| Factory | 31,824 (10.62) | 9,823 (8.52) | 21,992 (11.93) | 9 (18.00) |  |
| Administrative/Managerial/Sales | 18,819 (6.28) | 4,839 (4.20) | 13,976 (7.58) | 4 (8.00) |  |
| Professional/  Technical | 7,736 (2.58) | 1,890 (1.64) | 5,846 (3.17) | 0 (0.00) |  |
| Unemployed/Retired | 61,000 (20.36) | 15,860 (13.76) | 45,131 (24.49) | 9 (18.00) |  |
| Housewife | 47,146 (15.73) | 20,013 (17.36) | 27,132 (14.72) | 1 (2.00) |  |
| Self-employed/Others | 11,179 (3.73) | 3,273 (2.84) | 7,904 (4.29) | 2 (4.00) |  |
| **Marital status, n (%)** |  |  |  |  |  |
| Married | 267,264 (89.20) | 102,986 (89.33) | 164,231 (89.11) | 47 (94.00) | <0.0001 |
| Widowed | 27,776 (9.27) | 11,301 (9.80) | 16,473 (8.94) | 2 (4.00) |  |
| Separated/  Divorced | 4,468 (1.49) | 969 (0.84) | 3,498 (1.90) | 1 (2.00) |  |
| Single | 121 (0.04) | 27 (0.02) | 94 (0.05) | 0 (0.00) |  |
| **Household income (yuan), n (%)** |  |  |  |  |  |
| <5000 | 30,372 (10.14) | 15,998 (13.88) | 14,369 (7.80) | 5 (10.00) | <0.0001 |
| 5000 – 19,999 | 147,195 (49.13) | 60,208 (52.23) | 86,962 (47.19) | 25 (50.00) |  |
| ≥20,000 | 122,062 (40.74) | 39,077 (33.90) | 82,965 (45.02) | 20 (40.00) |  |
| **Waist-hip ratio, mean (SD)** | 0.87 (0.07) | 0.87 (0.07) | 0.86 (0.07) | 0.87 (0.07) |  |
| **MET hours, n (%)** |  |  |  |  |  |
| <16.8 | 148,150 (49.44) | 53,272 (46.21) | 94,854 (51.47) | 24 (48.00) | <0.0001 |
| ≥16.8 | 151,479 (50.56) | 62,011 (53.79) | 89,442 (48.53) | 26 (52.00) |  |
| **BMI, n (%)** |  |  |  |  |  |
| <25 | 196,809 (65.68) | 77,486 (67.21) | 119,285 (64.72) | 38 (76.00) | <0.0001 |
| ≥25 | 102,819 (34.32) | 37,797 (32.79) | 65,011 (35.28) | 12 (24.00) |  |
| Missing | 1 (0.00) | 0 (0.00) | 1 (0.00) | 0 (0.00) | <0.0001 |
| **Smoking, n (%)** |  |  |  |  |  |
| Smoker | 15,141 (5.05) | 5,213 (4.52) | 9,914 (5.38) | 14 (28.00) | <0.0001 |
| Non-smoker | 284,488 (94.95) | 110,070 (95.48) | 174,382 (94.62) | 36 (72.00) |  |
| **Alcohol, n (%)** |  |  |  |  |  |
| Alcohol drinker | 108,979 (36.37) | 36,279 (31.47) | 72,677 (39.43) | 23 (46.00) | <0.0001 |
| Non-alcohol drinker | 190,650 (63.63) | 79,004 (68.53) | 111,619 (60.57) | 27 (54.00) |  |
| **Gum bleed, n (%)** |  |  |  |  |  |
| No | 186,633 (62.29) | 70,790 (61.41) | 115,806 (62.84) | 37 (74.00) | <0.0001 |
| Yes | 112,996 (37.71) | 44,493 (38.59) | 68,490 (37.16) | 13 (26.00) |  |
| **Parity, n (%)** |  |  |  |  |  |
| 0 | 1,365 (0.46) | 104 (0.09) | 1,261 (0.68) | 0 (0.00) | <0.0001 |
| 1 | 105,637 (35.26) | 27,422 (23.79) | 78,197 (42.43) | 18 (36.00) |  |
| 2 | 99,330 (33.15) | 42,852 (37.17) | 56,464 (30.64) | 14 (28.00) |  |
| ≥3 | 93,261 (31.13) | 44,891 (38.94) | 48,352 (26.24) | 18 (36.00) |  |
| Missing | 36 (0.01) | 14 (0.01) | 22 (0.01) | 0 (0.00) |  |
| **Pregnancy, n (%)** |  |  |  |  |  |
| 0 | - | - | - | - | <0.0001 |
| 1 | 27,386 (9.14) | 26,765 (23.22) | 620 (0.34) | 1 (2.00) |  |
| 2 | 78,036 (26.04) | 40,834 (35.43) | 37,193 (20.18) | 0 (0.00) |  |
| ≥3 | 194,159 (64.80) | 47,675 (41.35) | 146,482 (79.48) | 2 (4.00) |  |
| Missing | 48 (0.02) | 0 (0.00) | 1 (0.00) | 47 (94.00) |  |
| **Livebirths, n (%)** |  |  |  |  |  |
| 0 | 1,214 (0.41) | 0 (0.00) | 1,214 (0.66) | 0 (0.00) | <0.0001 |
| 1 | 104,047 (34.73) | 26,765 (23.22) | 77,280 (41.93) | 2 (4.00) |  |
| 2 | 96,042 (32.05) | 40,843 (35.43) | 55,199 (29.95) | 0 (0.00) |  |
| ≥3 | 98,279 (32.80) | 47,675 (41.35) | 50,603 (27.46) | 1 (2.00) |  |
| Nulligravid | - | - | - | - |  |
| Missing | 47 (0.02) | 0 (0.00) | 0 (0.00) | 47 (94.00) |  |
| **Spontaneous abortion, n (%)** |  |  |  |  |  |
| 0 | 272,424 (90.92) | 115,283 (100.00) | 157,141 (85.27) | 0 (0.00) | <0.0001 |
| 1 | 21,412 (7.15) | 0 (0.00) | 21,411 (11.62) | 1 (2.00) |  |
| 2 | 4,240 (1.42) | 0 (0.00) | 4,240 (2.30) | 0 (0.00) |  |
| ≥3 | 1,504 (0.50) | 0 (0.00) | 1,504 (0.82) | 0 (0.00) |  |
| Nulligravid | - | - | - | - |  |
| Missing | 49 (0.02) | 0 (0.00) | 0 (0.00) | 49 (98.00) |  |
| **Induced abortion, n (%)** |  |  |  |  |  |
| 0 | 142,348 (47.51) | 115,283 (100.00) | 27,065 (14.69) | 0 (0.00) | <0.0001 |
| 1 | 83,153 (27.75) | 0 (0.00) | 83,153 (45.12) | 0 (0.00) |  |
| 2 | 48,160 (16.07) | 0 (0.00) | 48,160 (26.13) | 0 (0.00) |  |
| ≥3 | 25,919 (8.65) | 0 (0.00) | 25,918 (14.06) | 1 (2.00) |  |
| Nulligravid | - | - | - | - |  |
| Missing | 49 (0.02) | 0 (0.00) | 0 (0.00) | 49 (98.00) |  |
| **Stillbirth, n (%)** |  |  |  |  |  |
| 0 | 282,538 (94.30) | 115,283 (100.00) | 167,255 (90.75) | 0 (0.00) | <0.0001 |
| 1 | 13,174 (4.40) | 0 (0.00) | 13,174 (7.15) | 0 (0.00) |  |
| 2 | 2,762 (0.92) | 0 (0.00) | 2,762 (1.50) | 0 (0.00) |  |
| ≥3 | 1,105 (0.37) | 0 (0.00) | 1,105 (0.60) | 0 (0.00) |  |
| Nulligravid | - | - | - | - |  |
| Missing | 50 (0.02) | 0 (0.00) | 0 (0.00) | 50 (100.00) |  |

BMI = Body mass index, MET = Metabolic equivalent of task value, RA = Rheumatoid arthritis

**Table 2.** Stratified effect estimates of the association between spontaneous abortion, induced abortion, and stillbirth with rheumatoid arthritis

|  | **Spontaneous abortion** | | | | | **Induced abortion** | | | | | **Stillbirth** | | | | |
| --- | --- | --- | --- | --- | --- | --- | --- | --- | --- | --- | --- | --- | --- | --- | --- |
|  | Ever | 1 | 2 | ≥3 | Per additional | Ever | 1 | 2 | ≥3 | Per additional | Ever | 1 | 2 | ≥3 | Per additional |
| **Region** |  |  |  |  |  |  |  |  |  |  |  |  |  |  |  |
| Rural | 1.03 (0.92 – 1.15) | 1.04 (0.92 – 1.18) | 0.79 (0.59 – 1.05) | 1.63 (1.16 – 2.29)* | 1.04 (0.96 – 1.12) | 1.19 (1.09 – 1.29)* | 1.15 (1.04 – 1.27)* | 1.25 (1.11 – 1.41)* | 1.21 (1.04 – 1.40)* | 1.09 (1.04 – 1.13)* | 1.00 (0.87 – 1.16) | 1.06 (0.90 – 1.24) | 0.93 (0.68 – 1.28) | 0.68 (0.40 – 1.15) | 0.96 (0.87 – 1.06) |
| Urban | 1.19 (1.07 – 1.33)* | 1.15 (1.02 – 1.30)* | 1.37 (1.06 – 1.78)* | 1.34 (0.86 – 2.07) | 1.14 (1.06 – 1.23)* | 1.08 (1.01 – 1.15)* | 1.05 (0.98 – 1.14) | 1.09 (1.00 – 1.18) | 1.13 (1.02 – 1.25)* | 1.04 (1.01 – 1.07)* | 1.13 (0.99 – 1.29) | 1.16 (1.00 – 1.34)* | 1.02 (0.71 – 1.45) | 0.91 (0.45 – 1.86) | 1.07 (0.97 – 1.18) |
| **Income** |  |  |  |  |  |  |  |  |  |  |  |  |  |  |  |
| <5000 | 1.01 (0.83 – 1.23) | 1.02 (0.81 – 1.27) | 0.95 (0.60 – 1.51) | 1.09 (0.57 – 2.08) | 1.01 (0.88 – 1.15) | 1.16 (0.99 – 1.35) | 1.09 (0.90 – 1.31) | 1.16 (0.92 – 1.47) | 1.32 (1.01 – 1.72)* | 1.09 (1.01 – 1.18)* | 1.07 (0.83 – 1.37) | 1.07 (0.80 – 1.42) | 1.10 (0.67 – 1.83) | 1.02 (0.47 – 2.21) | 1.04 (0.88 – 1.22) |
| 5000 – 19,999 | 1.11 (1.00 – 1.24)* | 1.14 (1.01 – 1.28)* | 0.82 (0.61 – 1.10) | 1.67 (1.18 – 2.37)* | 1.08 (1.00 – 1.17)* | 1.09 (1.01 – 1.18)* | 1.06 (0.97 – 1.16) | 1.13 (1.02 – 1.25)* | 1.12 (0.99 – 1.26) | 1.05 (1.01 – 1.08)* | 1.10 (0.96 – 1.26) | 1.16 (1.00 – 1.35)* | 0.90 (0.64 – 1.28) | 0.74 (0.41 – 1.32) | 1.01 (0.92 – 1.12) |
| ≥20,000 | 1.21 (0.98 – 1.28) | 1.05 (0.90 – 1.22) | 1.44 (1.06 – 1.95)* | 1.55 (0.90 – 2.67) | 1.13 (1.02 – 1.24)* | 1.13  (1.04 – 1.23)* | 1.11 (1.02 – 1.22)* | 1.14 (1.02 – 1.26)* | 1.16 (1.01 – 1.32)* | 1.05 (1.01 – 1.10)* | 1.03 (0.87 – 1.22) | 1.06 (0.89 – 1.28) | 1.03 (0.67 – 1.57) | 0.53 (0.20 – 1.45) | 0.99 (0.87 – 1.13) |
| **MET** |  |  |  |  |  |  |  |  |  |  |  |  |  |  |  |
| <16.8 | 1.11 (1.00 – 1.22)* | 1.13 (1.01 – 1.25)* | 0.95 (0.74 – 1.22) | 1.41 (1.01 – 1.99)* | 1.08 (1.00 – 1.15)* | 1.10 (1.03 – 1.18)* | 1.06 (0.98 – 1.15) | 1.14 (1.04 – 1.24)* | 1.15 (1.03 – 1.28)* | 1.05 (1.02 – 1.09)* | 1.08 (0.96 – 1.21) | 1.14 (1.00 – 1.30)* | 0.92 (0.70 – 1.22) | 0.66 (0.40 – 1.09) | 1.00 (0.92 – 1.08) |
| ≥16.8 | 1.10 (0.96 – 1.25) | 1.05 (0.91 – 1.21) | 1.19 (0.88 – 1.61) | 1.66 (1.08 – 2.56)* | 1.10 (1.01 – 1.21)* | 1.13 (1.04 – 1.24)* | 1.13 (1.03 – 1.24)* | 1.13 (1.00 – 1.26)* | 1.16 (1.01 – 1.33)* | 1.05 (1.01 – 1.10)* | 1.08 (0.90 – 1.28) | 1.05 (0.86 – 1.27) | 1.14 (0.74 – 1.75) | 1.91 (0.56 – 2.55) | 1.06 (0.93 – 1.20) |
| **BMI** |  |  |  |  |  |  |  |  |  |  |  |  |  |  |  |
| <25 | 1.11  (1.01 – 1.23)* | 1.10 (0.98 – 1.23) | 1.05 (0.82 – 1.35) | 1.50 (1.06 – 2.11)* | 1.09 (1.02 – 1.17)* | 1.14 (1.07 – 1.22)* | 1.12 (1.03 – 1.21)* | 1.15 (1.05 – 1.26)* | 1.22 (1.09 – 1.36)* | 1.07 (1.03 – 1.10)* | 1.06 (0.93 – 1.21) | 1.12 (0.97 – 1.29) | 0.93 (0.69 – 1.25) | 0.72 (0.43 – 1.20) | 1.00 (0.91 – 1.09) |
| ≥25 | 1.10 (0.97 – 1.24) | 1.09 (0.95 – 1.25) | 1.00 (0.74 – 1.36) | 1.50 (0.98 – 2.30) | 1.08 (0.99 – 1.17) | 1.08 (1.00 – 1.17) | 1.06 (0.96 – 1.16) | 1.12 (1.01 – 1.25)* | 1.08 (0.95 – 1.23) | 1.04 (1.00 – 1.08) | 1.06 (0.91 – 1.24) | 1.09 (0.92 – 1.29) | 1.03 (0.71 – 1.51) | 0.78 (0.38 – 1.60) | 1.02 (0.91 – 1.14) |
| **Smoking** |  |  |  |  |  |  |  |  |  |  |  |  |  |  |  |
| Smoker | 0.82 (0.61 – 1.11) | 0.72 (0.50 – 1.03) | 1.08 (0.58 – 2.00) | 1.39 (0.60 – 3.23) | 0.96 (0.79 – 1.17) | 0.90 (0.74 – 1.08) | 0.83 (0.66 – 1.04) | 0.93 (0.71 – 1.21) | 1.05 (0.79 – 1.40) | 1.00 (0.92 – 1.10) | 1.11 (0.80 – 1.55) | 1.05 (0.73 – 1.52) | 1.76 (0.90 – 3.43) | 0.42 (0.06 – 3.09) | 1.08 (0.85 – 1.36) |
| Non-smoker | 1.13 (1.05 – 1.23)* | 1.13  (1.04 – 1.24)* | 1.02 (0.83 – 1.25) | 1.51 (1.14 – 2.00)* | 1.10 (1.04 – 1.16)* | 1.13 (1.07 – 1.19)* | 1.11 (1.04 – 1.18)* | 1.15 (1.07 – 1.24)* | 1.16 (1.06 – 1.27)* | 1.06 (1.03 – 1.08)* | 1.06 (0.96 – 1.18) | 1.11 (1.00 – 1.25) | 0.91 (0.71 – 1.17) | 0.76 (0.50 – 1.17) | 1.00 (0.93 – 1.08) |
| **Alcohol** |  |  |  |  |  |  |  |  |  |  |  |  |  |  |  |
| Alcohol drinker | 1.14 (1.01 – 1.30)* | 1.12 (0.97 – 1.29) | 1.13 (0.83 – 1.54) | 1.48 (0.95 – 2.31) | 1.11 (1.01 – 1.21)* | 1.06 (0.97 – 1.16) | 1.04 (0.94 – 1.14) | 1.08 (0.97 – 1.21) | 1.11 (0.97 – 1.26) | 1.04 (1.00 – 1.08) | 1.10 (0.91 – 1.32) | 1.14 (0.94 – 1.39) | 1.19 (0.76 – 1.87) | 0.15 (0.02 – 1.08) | 1.01 (0.88 – 1.17) |
| Non-alcohol drinker | 1.08 (0.98 – 1.20) | 1.08 (0.97 – 1.20) | 0.97 (0.75 – 1.24) | 1.51 (1.08 – 2.12)* | 1.07 (1.00 – 1.15) | 1.14 (1.06 – 1.22)* | 1.11 (1.03 – 1.20)* | 1.16 (1.06 – 1.27)* | 1.18 (1.06 – 1.33)* | 1.06 (1.03 – 1.10)* | 1.05 (0.94 – 1.18) | 1.10 (0.96 – 1.25) | 0.91 (0.69 – 1.20) | 0.91 (0.59 – 1.40) | 1.01 (0.93 – 1.09) |

BMI = Body mass index, MET = Metabolic equivalent of task value

Models excludes nulligravid women. Women without spontaneous abortion, induced abortion, and stillbirth, as appropriate, were used as reference.

**Region:** Adjusted for age, province, education, occupation, income, physical activity, body mass index, alcohol use, smoking, gum bleed, hypertension diagnosis, diabetes diagnosis, livebirths, and stillbirths, spontaneous abortion, induced abortion, as appropriate.

**Income:** Adjusted for age, province, region, education, occupation, physical activity, body mass index, alcohol use, smoking, gum bleed, hypertension diagnosis, diabetes diagnosis, livebirths, and stillbirths, spontaneous abortion, induced abortion, as appropriate.

**Metabolic equivalent of task value:** Adjusted for age, province, region, education, occupation, income, body mass index, alcohol, smoking, gum bleed, hypertension diagnosis, diabetes diagnosis, livebirths, and stillbirths, spontaneous abortion, induced abortion, as appropriate.

**Body mass index:** Adjusted for age, province, region, education, occupation, income, physical activity, alcohol use, smoking, gum bleed, hypertension diagnosis, diabetes diagnosis, livebirths, and stillbirths, spontaneous abortion, induced abortion, as appropriate

**Smoking:** Adjusted for age, province, region, education, occupation, income, physical activity, body mass index, alcohol use, gum bleed, hypertension diagnosis, diabetes diagnosis, livebirths, and stillbirths, spontaneous abortion, induced abortion, as appropriate.

**Alcohol:** Adjusted for age, province, region, education, occupation, income, physical activity, body mass index, smoking, gum bleed, hypertension diagnosis, diabetes diagnosis, livebirths, and stillbirths, spontaneous abortion, induced abortion, as appropriate.
